# Gigantea: Uncovering New Functions in Flower Development

**DOI:** 10.3390/genes11101142

**Published:** 2020-09-28

**Authors:** Claudio Brandoli, Cesar Petri, Marcos Egea-Cortines, Julia Weiss

**Affiliations:** 1 Genética Molecular, Instituto de Biotecnología Vegetal, Edificio I+D+I, Plaza del Hospital s/n, Universidad Politécnica de Cartagena, 30202 Cartagena, Spain; claudio.brandoli@gmail.com (C.B.); marcos.egea@upct.es (M.E.-C.); 2 Instituto de Hortofruticultura Subtropical y Mediterránea-UMA-CSIC, Departamento de Fruticultura Subtropical y Mediterránea, 29750 Algarrobo-costa, Málaga, Spain; cesar.petri@csic.es

**Keywords:** Gene ontology, molecular function, cellular localisation, biological function, circadian clock, flowering time, flower development, floral scent

## Abstract

*GIGANTEA (GI)* is a gene involved in multiple biological functions, which have been analysed and are partially conserved in a series of mono- and dicotyledonous plant species. The identified biological functions include control over the circadian rhythm, light signalling, cold tolerance, hormone signalling and photoperiodic flowering. The latter function is a central role of *GI*, as it involves a multitude of pathways, both dependent and independent of the gene *CONSTANS(CO)*, as well as on the basis of interaction with miRNA. The complexity of the gene function of *GI* increases due to the existence of paralogs showing changes in genome structure as well as incidences of sub- and neofunctionalization. We present an updated report of the biological function of *GI*, integrating late insights into its role in floral initiation, flower development and volatile flower production.

## 1. Introduction

GIGANTEA (GI) is a plant-specific nuclear protein, identified for the first time in *Arabidopsis thaliana* as a late flowering mutant [1]. Although six decades have passed since its discovery, its precise molecular function has not been completely elucidated. Only at the end of the XXth century was it possible to obtain information describing its chromosomal organization [2]. The mapping identified the genomic locus on chromosome 1, consisting of 14 exons and encoding for a protein of 1,173 amino acids [2,3]. The *GI* gene, which appeared early in land plants, is present in a single copy in most plants, such as Arabidopsis or rice [4], while in *Solanaceae* it is found in two or three copies [5]. Evolutionary phylogenetic analysis of the gene has shown that *GI* in Petunia and in general in *Solanaceae*, is grouped separately from the clade of *Brassicaceae*, *Rosaceae* and *Fabaceae*. This indicates an evolutionary departure that appears to be specific to plant families. Further gene duplications have been found in the subclades of tomato, *Nicotiana benthamiana* and *Petunia inflata* [5].

Recent studies carried out in Petunia [6] support the hypothesis that the structural evolution of the main circadian clock genes such as *PhGI*, occurred through changes in the number of paralogues via gene duplications. Further changes have occurred in the gene structure and in the coding region, including both deletions and substitutions, depending on the species and paralog.

This review gives an overview of the molecular functions, cellular localisation and biological functions of GI paralogs, described in model plants as well as crops of agronomic importance. 

## 2. The Cellular Localisation of Gigantea

The cellular localisation of *GI* in Arabidopsis was identified by constitutively expressing *GI-GFP* both transiently in protoplasts and transgenic plants. Fluorescent microscopy analysis revealed that GI protein is predominantly present in the nucleus of some cell types, forming nuclear bodies [7,8]. Importantly, GI proteins lack a nuclear localisation signal. To better understand the nature of these formations, specific sub-nuclear marker-proteins of different compartments were used, directed specifically to nucleoli, spliceosomes, heterochromatin beams and Cajal bodies. GI was not localized in any of the aforementioned nuclear compartments, showing that GI does not play any role in the processes of protein degradation, pre-mRNA splicing and biogenesis of rRNA and snRNP. The evening complex gene *EARLY FLOWERING 4 (ELF4)* was found to sequester GI from the nucleoplasm, where it binds to the *CO* promoter, to discrete nuclear bodies [8]. This process is induced under long days in Arabidopsis. ELF4 protein synthesis oscillates during the day and consequently does the formation of GI nuclear bodies, peaking at night, and it is proposed that GI can then be released in the morning, avoiding the necessity for de novo GI synthesis [8].

GI is also localized in the cytosol where it stabilizes the F-box protein ZEITLUPE (ZTL) through heterodimerization. Under blue light, GI binds to the LOV (light, oxygen or voltage sensing) domain of ZTL, thus ensuring a robust and accurate oscillation of its target proteins [9]. It was proposed that sequestration of GI by ZTL to the cytosol, regulates the nuclear pool of GI and controls thereby the distribution of GI between nucleus and cytosol as well as its protection from degradation [10]. Degradation in the nucleus coincides with the high accumulation of *CONSTITUTIVE PHOTOMORPHOGENIC 1*(COP1) and clock-associated protein ELF3. COP1 is an E3 ubiquitin-ligase, and *ELF3* allows COP1 to interact with GI leading to GI degradation under short day conditions. Thus, the destabilisation of GI through COP1 and ELF3 plays an important role in the modulation of circadian rhythms and regulation of flowering transition in Arabidopsis [11].

## 3. The Molecular Functions of Gigantea

### 3.1. Gigantea Coordinates Light Signalling, Protein Degradation and Transcription of the Circadian Clock 

**Figure 1:**
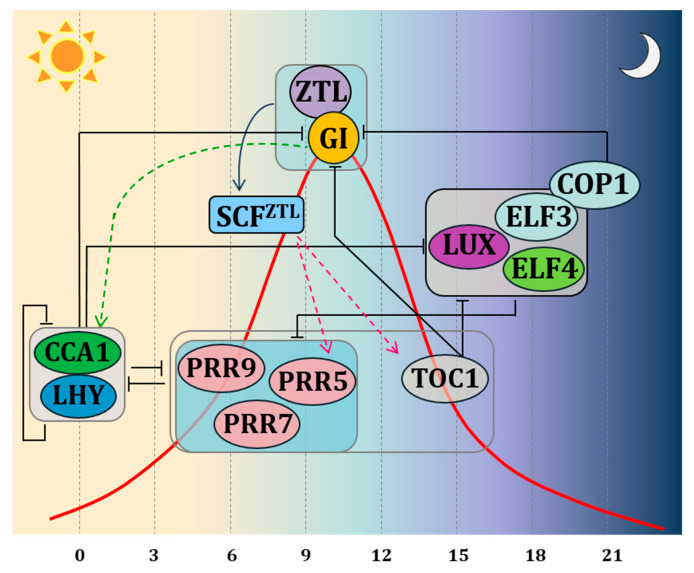
Schematic representation of the clock model indicating interactions by autoregulatory feedback loops via transcriptional and post-translational activation and repression. The clock components are depicted according to their peak-time expression from left to right. Black bars indicate repression. Green arrowheads lines indicate activation of transcription. Fucsia arrowheads lines indicate proteasomal degradation. The red curve represents the expression of GI during the day. TOC1 is protected during the day from ZTL-induced proteasome degradation thanks to the direct protein-protein interaction of GI with ZTL. During the evening, ELF3 promotes the proteasome degradation of GI through its interaction with COP1, thus promoting the ZTL-dependent TOC degradation. Boxes indicate sets of genes acting in coordination. The PRR box: PRR 5,7,9—these proteins are sequentially expressed during the day, and all repress LHY and CCA1; GI-ZTL box—protein-protein interaction leads to ZTL maturation; ELF3-ELF4-LUX complex box—protein complex peaking at dusk.

The most recent clock model, based on Arabidopsis, describes the endogenous clock as an intricate system of negative autoregulatory feedback loops interacting with each other via transcriptional and post-translational activation and repression [12] (Figure 1). Two MYB domain transcription factors, *CIRCADIAN CLOCK ASSOCIATED 1 (CCA1)* and *LATE ELONGATED HYPOCOTYL (LHY)* form the central loop together with *PSEUDO-RESPONSE REGULATOR 1 (PRR1)*, also known as *TIMING OF CAB EXPRESSION 1 (TOC1).* The morning loop is formed by *PSEUDO-RESPONSE REGULATORs* (*PRRs*) 9,7 and 5 [13]. The evening loop is composed of *EARLY FLOWERING 3 and 4 (ELF3 and ELF4)* and *LUX ARHYTHMO (LUX)*, together with *ZEITLUPE (ZTL)* [14]. *ELF* genes and *ZTL* control light input signals to the clock and the ability of plants to distinguish between different day lengths [2,9,15]. GI interacts with several of these clock genes, ensuring expression peaks, period length and amplitude of the different clock genes at specific times of the day. In Arabidopsis, *GI* transcript levels are controlled by the circadian clock with peak transcript levels at 8–10 h after dawn [2]. In *Petunia*, a lack of robust circadian rhythmicity under continuous darkness for *PhGI1* hints to the necessity of correct light signalling for oscillation [16].

One molecular function within the circadian oscillator complex consists of the binding of GI protein to ZEITLUPE (ZTL), a protein codified by a gene belonging to the evening complex. ZTL receives light inputs through its LIGHT, OXYGEN, VOLTAGE (LOV) domain, sensing blue-light, but it also has an F-box, targeting protein for degradation. ZTL degrades the central clock protein TOC1 [14]. GI is crucial for stabilizing and maintaining the oscillations of ZTL blue-light photoreceptor through direct protein protein interaction. In this connection, GI interacts with the blue light-sensitive domain of ZTL, stabilising it post-translationally under blue light [9,17]. In Arabidopsis, the expression of *ZTL* is uniform during 24 h, while protein levels oscillate [18]. In contrast, transcription of *PhCHL*, the Petunia ortholog of *ZTL*, is characterized by a significant rise at midday [16,19]. There are two paralogs of *GI* in *Petunia* × *hybrida*, *PhGI1* and *PhGI2*. The Silencing of *PhGI1* causes a significant prolongation of the rhythmic period of expression of *PhCHL* [16]. This suggests that coordination of PhCHL by PhGI does not only occur at the post-translational level, but that *PhGI* also coordinates the timing of expression of *PhCHL* during the day. However, it is not clear yet if this is a direct effect or happens as a result of modified levels of proteins involved in transcription such as PhTOC1.

According to the theory of the repression feedback circuits, the morning elements LHY and CCA1 heterodimerize and repress the expression of *TOC1* and the evening complex members as *GI*, *LUX*, *ELF3*, *ELF4*. PRR9, PRR7 and PRR5, which in turn, when expressed, repress the expression of *CCA1* and *LHY*. In the evening, *TOC1* represses all of the previously expressed components. The molecular interaction between GI and ZTL on one side and ZTL and TOC1 on the other side, predicts changes in the expression pattern of clock genes affected by TOC1 protein levels. Indeed, experiments carried out by Fowler et al. (1999) in Arabidopsis demonstrated that under both long days (LDs) and short days (SDs), a mutation in the *GI* locus affects the *CCA1* and *LHY* gene expression. On the other hand, over-expression or mutations of *CCA1* and *LHY* disrupt the *GI* expression. In agreement with these results, the double mutant of *LHY* and *CCA1* shows an overabundance of the transcription of *GI* [7]. These results led to the conclusion that *GI* operates in a feedback loop for adjusting and maintaining the length of the clock period. The involvement of *GI* in circadian clock control was confirmed in rice, were *OsGI* controls amplitude and circadian rhythm phases of global gene expression under natural field conditions [4].

### 3.2. Flowering Time and Photoperiod Related Molecular Functions

In Arabidopsis, photoperiod, causing the transition from the vegetative phase to flowering, is regulated by the diurnal expression of *CONSTANS* (*CO*) [20,21]. It has been shown that during long days (LD), light stabilises CO protein. In Arabidopsis, the expression of *CO* coinciding with the period of light leads to the activation of *FLOWERING LOCUS T* (*FT*) gene. On the other hand, under short day (SD) conditions, the peak expression of *CO* occurs after sunset because the *CO* protein is not sufficiently stabilized by light [22]. The transcription of *CO* is repressed during sunrise, due to the activity of the *CYCLING DOF FACTOR 1* (*CDF1*) transcriptional repressor bound to the *CO* promoter.

**Figure 2:**
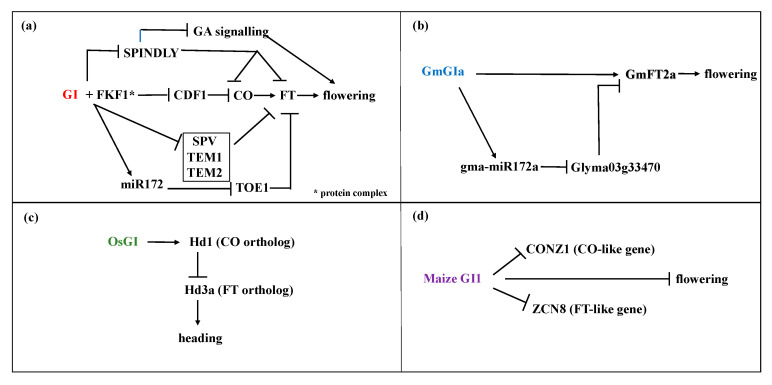
Models of GIGANTEA dependent flowering time regulation in (**a**) Arabidopsis, (**b**) soybean, (**c**) rice (**d**) and maize under long day condition. Regulation of flowering through GI differs between LD and SD plants. In contrast to LD plant Arabidopsis, *OsGI* inhibits flowering in rice, an SD plant, because the *FT* ortholog *Hd3a* is repressed by *Hd1*, the *CO* ortholog.

GIGANTEA plays a key role in this flowering regulation pathway. Figure 2 illustrates the involvement of GI in flowering time control in Arabidopsis as well as in other model plants. Although several paralogs of GI exist in different species, i.e., maize, thus far, the existing models on flowering time control represent the function of only one of the paralogs, and further research may elucidate further functions of GI in other plants. In Arabidopsis, an enzymatic complex is formed thanks to a direct protein-protein interaction between GI and FLAVIN-BINDING, KELCH REPEAT, F BOX protein 1 (FKF1), through its FKF1 LOV (Light, Oxygen or Voltage) domain [23]. This complex mediates the degradation of *CYCLING DOF FACTOR 1* (*CDF1*), a main *CO* repressor. This complex is strictly dependent on light, being the expression of *GI* under the control of the circadian clock. Therefore, towards the middle of the day, when the accumulation of GI along with FKF1 reaches the peak, the *DOF*-degradation complex is repressed, leading to an increase in the transcription of *CO* and, therefore, to the transcription of *FLOWERING LOCUS T* (*FT)* [23,24]. This does not happen in short days in Arabidopsis, as the GI accumulation peak occurs about three hours earlier than that of FKF1. This prevents the formation of the *DOF*-degradation complex and the consequently reduced abundance of the *CO* transcript.

Another light-dependent control of flowering through GIGANTEA involves *SPINDLY* (*SPY*). GI inhibits SPY in a light dependant manner. At the same time, *SPY* represses the expression of *CO* and *FT*, as observed in *spy-4* plants, where the late-flowering phenotype of *gi-2* plants was abolished due to a partial suppression of abundance reduction in *CO* and *FT* RNA [25]. Furthermore, SPY also affects flowering by inhibiting GA signaling, required for flower promotion [26]

Additionally, *GI* can regulate the expression of *FT* independently of *CO*. It inhibits the *FT* transcriptional repressors such as *SHORT VEGETATIVE PHASE* (*SVP*), *TEMPRANILLO 1* (*TEM1*) and *TEMPRANILLO 2* (*TEM2*). GI alters their stability or neutralises their repressor effect by blocking their access to the FT promoter region. Likewise, GI binds their specific target regions on the *FT* promoter [27], thus affecting the abundance of the FT transcripts.

*GI* regulates *FT* expression and photoperiodic flowering, independent of *CO*, via its interaction with a microRNA. The *miRNA172* inhibits the expression of the main transcriptional repressors of *FT*, *TARGET OF EAT 1* (*TOE1*) and *APETALA 2* (*AP2*). It was demonstrated that *miRNA172* processing is positively regulated in the presence of GI protein through an unclear molecular interaction [28]. Growth of plants under natural conditions, i.e., with photo and thermoperiod, shows that it has a major impact in the coordination of FT, suggesting that GI may have a role in temperature and light coordination [29].

Many *GIGANTEA* orthologs have been described in the last decades, in a wide range of plant species, from gymnosperms to mono y dicotyledon angiosperms. Many of these have similar expression patterns to those of Arabidopsis *GI*, suggesting broad conservation of the photoperiodic flowering regulation mechanisms (Figure 2) [30,31,32,33,34,35,36]. Indeed, the *GI* ortholog of soybean, *GmGIa*, controls flowering time by inducing the expression of the soybean florigen gene ortholog *GmFT2a* [37]. Among three haplotypes (*H1*, *H2*, *H3*) of *GmGIa*, *H1* rescues the late-flowering phenotype of *gi-2* in Arabidopsis, while *H2* and *H3* delay flowering in transgenic Arabidopsis with a wild type background. This diversification was proposed to be related to flowering time adaptation during soybean domestication [38]. Similar to Arabidopsis, *GmGIa* also positively regulates *gma-miR172a*, which in turn represses *Glyma03g33470*, leading to upregulation of *FT*, *AP2* and *LFY* and early flowering [39]. 

Wheat is a long-day (LD) plant and one *GIa* ortholog *TaGI1*, has been described. It shares 63% identity with *AtGI* and a superior homology to *GI* from grasses such as rice and barley [30]. Overexpression of *TaGI1* alters flowering time in wheat, resulting in early flowering both under LD and SD [30]. In barley, an upregulation of *HvGI* observed in *HvELF3* mutants results in an early flowering phenotype [40].

Conservation of GI function upon flowering time is also observed in short-day (SD) plants. The comparison of the sequences between Arabidopsis *GI* and its ortholog in *Oryza sativa GI* (*OsGI*) reveals 67% of identity as well as conservation of its nuclear localisation [41,42]. In rice, a quantitative SD plant, over-expression of the *GIGANTEA* gene (*OsGI*) under LD and SD leads to a delay in flowering time with a more pronounced effect under SD [43]. Rice overexpressing *OsGI* and under LD condition, show increased expression of the *CO* ortholog *Hd1*, while the expression of *Hd3a*, the *FT* ortholog, was reduced. Thus, a reverse regulation of *Hd3a* by *Hd1* occurs in rice under LD conditions. Opposite to Arabidopsis, *OsGI* acts as a flowering time suppressor [43,44,45]. However, under SD conditions *Hd1* promotes heading coinciding with increased transcript levels of *Hd3a*. This suggests that the regulatory network of the key flowering genes is conserved among rice and Arabidopsis [44]. Likewise, in the SD and C4 plant maize, which has two circadian regulated *GI* paralogs, *Zmgi1* mutants flower earlier than non-mutant plants in LD photoperiods, which is the opposite to the late-flowering gi mutants from Arabidopsis, but not in SD photoperiods [36]. The flowering time control mechanism also involves FT-like floral activator gene Centroradialis8 (ZCN8) and the CONSTANS-like flowering regulatory gene Constans of Zea mays1 (CONZ1), which are both upregulated in the Zmgi1 mutant, confirming the existence of the conserved network of the three key regulatory genes *Zmgi1, CONZ1, ZCN8* in maize, but gene interaction is photoperiod dependent and differs whether the species is a LD or SD plant. 

*Petunia* × *hybrida* has two *GI* paralogs, *PhGI1* and *PhGI2*. Downregulation of *PhGI1* by interference RNA does not induce late flowering, but a flowering time effect of *PhGI2* cannot be ruled out [16]. In the perennial poplar, three *GI-like* genes *PagGIa*, *PagGIb* and *PagGIc* were identified and overexpressed in Arabidopsis wild type ecotype Columbia-0 (Col-0), leading to early flowering [46]. Recent work in white lupin has identified *LaGI* as a major QTL involved in flowering time [47]. As the single copy gene *MpGI* coordinates phase transition into reproductive development in the basal angiosperm *Marchantia polymorpha* [48]. Indeed, loss of *MpGI* causes complete loss of long-day dependent phase transition. We can conclude that that *GI* plays a key role in flowering time in a variety of species.

### 3.3. Light Signalling Related Molecular Functions

Photoreceptors, such as phytochromes, phototropins, cryptochromes and UV-light photoreceptors, control light-induced plant development by the integration of light cues from the environment, such as quality, intensity and duration. Specific wavelength inputs are transformed into physiological signals, a process called photomorphogenesis [49,50]. Cryptochromes and phototropins absorb mainly the blue spectrum (B, λ = 400–499 nm) and are involved in the regulation of flowering time, inhibition of hypocotyl growth and phototropism [51]. Five members of phytochromes (Phy) exist in Arabidopsis, from PhyA to PhyE [42,52]. They act as red (R, λ = 660 nm; PhyB-E) and far-red (FR, λ = 730 nm; PhyA) photoreceptors. PhyA mediates two distinct photobiological responses: The very-low-fluence response (VLFR) and the high-irradiance response (HIR) [49]. Arabidopsis *gi*-mutants, grown under saturated R light condition, have shown an elongated hypocotyl compared to the wild type and little or no change in responsiveness to continuous FR light [42], indicating that *GI* appears to be a positive regulator of *PhyB* signalling during seedling de-etiolation [25]. Considering also that neither the genes nor the abundance of PhyA-B proteins are affected in *gi*-mutants, it appears that *GI* works downstream of PhyA-B, after their migration to the nucleus in response to light, where GI is constitutively localised. Additional data regarding *gi*-mutants grown in FR light condition revealed low VLFR levels, deficient cotyledon unfolding and a low seed germination index. These phenotypes were rescued through *GI* over-expression, demonstrating that *GI* plays a role in the PhyA signalling [53]. The *gi*-mutant seedlings also exhibited a long hypocotyl phenotype when grown under blue light, proving to have a role also in the signalling of blue light [50].

### 3.4. Hormone Signalling and Stress-Response Related Molecular Functions

One of the plant hormones, whose signalling interacts with GI, is gibberelins. In Arabidopsis, GI affects the growth of the hypocotyl through the interaction with *SPINDLY (SPY)*, a gene involved in the regulation of gibberellin signalling. *SPY* is, in fact, a negative regulator of gibberellin signalling and an inhibitor of hypocotyl lengthening [25,26]. GI interacts with a protein-protein interaction domain of SPY, consisting of 10 tetratricopeptide repeats (TPRs). Three pathways are controlled through SPY-GI interaction: Flowering, circadian cotyledon movements, and hypocotyl elongation. In the case of the flowering pathway, the reduction of *CO* and *FT* RNA abundance in *gi* plants is partially suppressed in the *spy* mutant. 

GI also interacts with gibberellin signalisation through its interaction with REPRESSOR OF ga1-3 (RGA), a DELLA protein. Under LD, warmer temperatures induce accumulation of GI, which functions as thermostabiliser of RGA, which leads to the attenuation of thermomorphogenesis mediated through PHYTOCHROME INTERACTING FACTOR 4 (PIF4). Under SD and lower GI levels, RGA is degraded through the gibberellic acid-mediated ubiquitination-proteasome pathway [54,55]. An effect of warm temperatures on GI accumulation was concluded from observations on GI-deficient *gi-2* mutants, which showed hypocotyl overgrowth at 28 °C but not at 23 °C. Overgrowth at warmer temperatures was related to an increased auxin action, as both *YUCCA8 (YUC8*), an auxin biosynthetic enzyme, and *SMALL AUXIN UPREGULATED RNA 22 (SAUR22)*, an auxin-responsive protein, were upregulated in *gi-2* mutants at warmer temperatures [54]. 

The Arabidopsis mutant *abz126* bears a T-DNA insertion into the ninth exon of the *GIGANTEA* (*GI*) gene, resulting in a loss-of-function of *GI*, leading to long petioles, tall plant height, many rosette leaves and late flowering, as well as insensitivity to paclobutrazol and brassinolide, indicating an association of *GI* with brassinosteroid hormone signalling [56].

Interaction between GI and stress response exists concerning tolerance to salt. Under normal conditions, GI interacts with the protein kinase SALT OVERLY SENSITIVE 2 (SOS2), thus preventing its interaction with the Na+/H+ anti-porter SOS1 to promote Na^2+^ export and salt tolerance. Under saline conditions, GI is degraded by the proteasome. Due to GI degradation under salt stress, SOS2 interacts with SOS3 and this protein kinase complex then activates SOS1, salt tolerance and retardation of flowering [57,58]. 

Opposite to salt stress, drought stress accelerates flowering time, and *GI* was found to be a key component mediating drought response in Arabidopsis. One proposed mode of action predicts that *GI* may regulate chromatin accessibility and/or interfere with repressor activity at the florigen promoters in a plant stress hormone abscisic acid (ABA) dependent manner [59].

*GI* also has molecular functions involved in freezing tolerance. *GI* activates the expression of *CO* and *FT*, the key floral regulators, by facilitating the degradation of a family of CDF1, which acts as transcriptional repressors. In Arabidopsis and tomato, in a *gi*-mutant background, increased stability and accumulation of CDF proteins and higher transcript levels of stress-responsive genes, including *COR15a*, *RD29A* and *ERD1*, were observed as well as an increased level of protection against cold stress [60,61]. Therefore, proposed that, in addition to flowering, the regulation of CDFs through GI, influences responses to freezing temperatures [61]. 

## 4. Biological Functions of Gigantea

The described molecular interactions of GI as well as its cellular location, result in a wide range of biological functions analysed in Arabidopsis. Part of these functions were found to be conserved in many other plant species, both monocots and dicots. Biological functions include circadian clock regulation, light signalling, flowering time control, which were already described in detail under molecular functions, as well as chlorophyll accumulation, sugar metabolism, stress tolerance, vegetative growth, flower development and floral scent emission described below. 

### 4.1. Chlorophyll Accumulation

**Figure 3:**
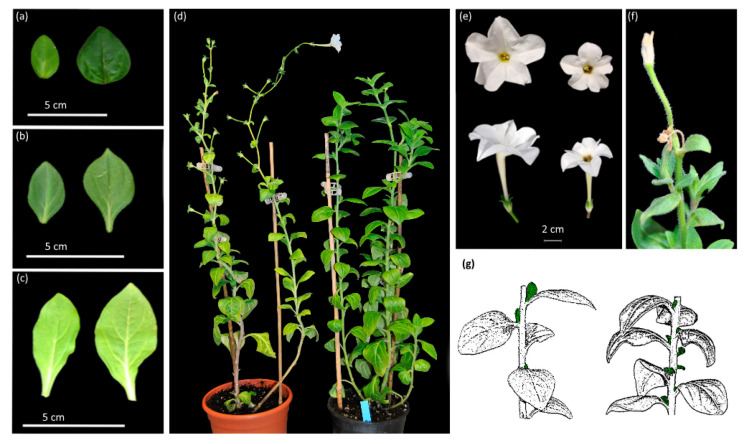
Phenotypic characteristics in *Petunia* × *hybrida* plants with loss of *GI1* function. Apical (**a**), median (**b**) and basal (**c**) leaves of wild type Petunia (left) compared to *iRNA*: *PhGI1* leaves (right). (**d**) Vegetative growth architecture of Petunia wild type (left) compared to the loss of *PhGI1* function (right). Petunia inflorescence appearance (**e**) of wild type (left) and *PhGI1* silenced line (right). Abortive flower (**f**,**g**) schematic representation of variations in internode length and number of axillary shoot meristems (indicated by the green colour) between wild type plants (left) and *iRNA*: *PhGI1* plants (right).

The analysis of different Arabidopsis *gi*-mutants hint to a *GI* function in chloroplast biogenesis and chlorophyll accumulation—factors essential for photosynthetic efficiency and therefore crop productivity. The Arabidopsis *gi-2* mutant is characterized by a reduced sensitivity to lincomycin, a chloroplast biogenesis inhibitor, and the mutant maintains high levels of photosynthetic proteins. In contrast, GI-overexpressing plants have variegated leaves, reduced photosynthetic protein levels and are sensitive to lincomycin [62]. A series of Arabidopsis mutants in Ler background (*gi-3,4,5*) showed a significantly higher chlorophyll content in seedlings [62]. RNA interference of one GI paralogue in *Petunia* × *hybrida*, *PhGI1*, leads to leaves with a greener appearance in the denser apical region coinciding with a progressive increase in chlorophyll content compared to the wild type [63] (Figure 3a–c). These results thus point to a general role of GI in chlorophyll homeostasis.

### 4.2. Sugar and Starch Metabolism

Sugars function in plants as a source of energy and as a signal, among others, in the circadian regulation of flowering [64]. The Arabidopsis circadian system was described to be sensitive to sucrose in the dark, indicating feedback between metabolism and circadian clock, and it was predicted that *GI* is required for the full response of the circadian clock to sucrose [65]. Indeed, GI protein is stabilized by sucrose in the night, and this mechanism requires interaction with ZTL [66].

Furthermore, the Arabidopsis *gi-3* mutant is characterized by an enhanced freezing sensitivity, and this was related to a reduction in soluble sugar content in leaves. Transcript levels of the cold-responsive gene *RD29A* and abscisic acid-responsive gene *RAB18* were not affected in this mutant, indicating a direct connection between *GI* gene function and sucrose metabolism [67]. In contrast to this observation, field-grown rice plants carrying a null mutation in the rice homolog *OsGI* showed a significantly increased sucrose and starch content in the leaves at most time points [4]. Increased starch content was also observed in *gi-1, 2 and 3* alleles in Arabidopsis [68]. A recent proteomic analysis of interactors of GI has identified TREHALOSE-6-PHOSPHATASE SYNTHASE 8 (TPS8) as a direct interactor [69]. This indicates a possible direct link between GI and sugar metabolism.

### 4.3. Stress Tolerance

Plants cope with environmental stresses by activating a series of specific metabolic pathways. Regarding low-temperature stress, they must avoid freezing injury that can occur in both intracellular and extracellular compartments, prevent chilling wounds and cell injuries that could cause tissue death. The response to cold temperatures implies alterations in the expression of genes, followed by increases in the levels of metabolites, including those known to have protective effects against the damaging effects of cold stress [70]. Plants have the ability to adapt to low temperatures by increasing their freezing tolerance through the gradual exposure to low but non-freezing temperatures, a process identified as cold acclimation [71]. Plant resistance to cold appears to be organ-specific [72]. This process is characterised by complex biochemical and physiological changes, including gene expression [73,74], enzyme activities [75], lipid membrane composition [76] and leaf ultrastructure modification [77]. Chilling injuries cause the collapse of deep cell layers and disruption of antioxidant activity amongst other effects [78]. The biological function of GI concerning freezing tolerance results from its interaction with CDF proteins. GI facilitates the degradation of CDF proteins. Arabidopsis *gi*-mutants accumulate CDFs accompanied by higher transcript levels of stress-responsive genes and increased cold stress [60,61]. Additionally, enhanced freezing sensitivity in *gi*-mutants is also related to a reduction in soluble sugar content [67]. Another direct biological function of GI is related to salt stress. This stress leads to proteasomal degradation of GI [57,58]. GI function in salt tolerance was found to be conserved in poplar, as overexpressing *PagGIs* in wild type (WT) Arabidopsis induces salt-sensitivity.

### 4.4. Vegetative Growth

Observations on plant development in Arabidopsis indicate that *GI* is a negative regulator of vegetative growth as can be inferred from its name as a mutant. Loss of function of *GI* effects hypocotyl growth, but it also results in long petioles, tall plant height and many rosette leaves [68]. The latter is proposed to be related to gibberellin signalling, as SPINDLY (SPY) protein, a negative regulator of gibberellin signalling in Arabidopsis and an inhibitor of hypocotyl elongation, interacts with GI protein [25]. Similarly, downregulation of the *GI1* paralog in Petunia by RNA interference results in bigger leaves (Figure 3a–c), an increased basal internode length, and an increased number of axillary meristems. Middle and apical internodes are reduced, indicating the existence of an acropetal gradient with opposite effects during the early stages of development and middle to late stages (Figure 3d,g). Similarly, downregulation of *PagGIs* in poplar by RNA interference leads to vigorous growth, and higher biomass [46].

### 4.5. Flower Development and Floral Scent Emission

A new role of *GI* in the development of reproductive organs has recently been described in *Petunia* × *hybrida*. Plants with loss of *GI1* function, grown under LD conditions, show a reduction in the total number of floral buds and smaller flowers than wild type. Flowers show a significant reduction in the corolla diameter as well as in the tube length (Figure 3e). There is an additional floral bud at the bifurcation point, where a given shoot that terminates in a flower and a new sympodial shoot separate (Figure 3f). The aborted flowers clearly show early development of carpel and stamen tissues, indicating that the senescence process occurs after the activation of the floral organ identity genes. These results indicate that *PhGI1* is a repressor of early flower senescence, a biological function that had not been described before. The significant reduction in the total number of flower buds also indicates an upstream effect related to the flower-meristem-identity genes *PETUNIA FLOWERING GENE (PFG)* and *ALF* (*ABERRANT LEAF AND FLOWER*) [79,80].

Among the main strategies that plants use to ensure entomophilic reproduction is the emission of floral scents in the form of volatile organic compounds (VOCs). Plant scents are mixtures of volatile lipophilic molecules of benzenoids/phenylpropanoids and fatty acid derivatives with low molecular weight and high vapor pressure at ambient temperature, synthesized in all plant organs, from roots to flowers [81,82]. The plant VOCs are classified into different classes according to their biosynthetic origins, such as: Terpenoids, benzenoids/phenylpropanoids, fatty acid derivatives, and amino acid derivatives as well as few other genus-specific species [83]. In vegetative organs, VOCs are part of the plant’s defence system and are mainly synthesised in glandular trichomes [84], single specialized cells [85] or tubes [86] from which they can sprout out in case of breakage. In many Angiosperms, the quantity and composition of the VOCs can fluctuate during the day, mainly in relation to the age of the flower. This rhythmic release of scent is also closely related to the flower hormonal regulation, circadian clock, flower and plant development, nutrient availability, temperature, humidity and general environmental conditions [87,88,89].

In recent decades, many studies on the production and regulation processes of VOCs in plants have been carried out, but few genes have been characterized involved in the regulation of scent production. The studies conducted by Verdonk et al., [90] in *Petunia* × *hybrida*, have identified a R2R3 MYB-type gene named *ODORANT1* (*ODO1*) which controls the synthesis of the precursor Phe in the shikimate pathway. The *Petunia* × *hybrida CHANEL* (*PhCHL*) gene, the ortholog of *Arabidopsis thaliana ZTL*, has been shown previously to play an important role in regulating both the timing and the quantity of volatile emissions [19]. Methyl benzoate is the dominant floral VOC in *Petunia hybrida*. Recent studies on *PhGI1* in *Petunia* × *hybrida* have revealed thus far unreported functions of this pleiotropic gene concerning the control over VOC emission. *PhGI1* loss of function plants exhibited a 20% reduction in the total emission of VOCs during 24 h. The circadian volatile emission pattern in the silenced lines remained unchanged. However, an important twist in the scent profile was observed, compared to the wild type and non-transgenic siblings with changes in the relative abundance of the *trans*-cinnamic acid derivatives benzyl alcohol, ethyl benzoate, benzyl benzoate and isoeugenol, indicating involvement of *GI* in the phenylalanine emission pathway, interfering in the rhythmic regulation of the VOC biosynthesis and their daily emission profile. Interestingly, a null mutation in the rice homolog *OsGI*, affects the production of several metabolites in the phenylpropanoid pathway, which was significantly increased, whereas the pool of Phe, the major chemical precursor in the pathway of phenylpropanoids, was significantly decreased [4]. These convergent results suggest the role of GI in the control of Phe and phenylpropanoids in plants.

## 5. Conclusions

GIGANTEA protein functions at multiple levels by interacting with genes involved in circadian rhythm, stress response, flowering time, light and hormone signaling, among others. It furthermore interacts with sugar metabolism and chlorophyll accumulation as well as vegetative growth. Some of these functions are found to be conserved across several plant species. The conserved function of GI in controlling flowering time was analyzed in detail in a wide range of species, including model plants such as Marchantia, Arabidopsis and Petunia, as well as in important crop plants such as rice, maize, barley or soya bean. Understanding this important trait is especially relevant in the case of plants with economic value as this developmental switch is relevant for the number of crop harvests per year or harvests under short vegetation time [91]. Both common and divergent patterns of molecular interaction involving GI can be observed, depending on whether the species belongs to long-day or short-day plants. Recent observations of *PhGI* in *Petunia hybrida* add yet another level of control of this gene on flower development, consisting in control over flower initiation, early flower senescence, flower size and volatile floral emission. In the latter case, PhGI seems to be involved both in the control of VOC quantity as well as in the fine-tuning of emission of VOCs belonging to the phenylalanine emission pathway. 

The existence of up to three paralogues in different plant species adds another level of complexity to the study of GI function. Recent studies carried out in Petunia [6] revealed differences in gene structure including coding regions, consisting of N-terminal deletions and C-terminal fragment insertions, depending on the specific paralog and species. Future studies on mutations in these specific paralogs may add further knowledge on the complex roles in plant development of this multifunctional protein.
